# The UCSC Genome Browser database: 2025 update

**DOI:** 10.1093/nar/gkae974

**Published:** 2024-10-26

**Authors:** Gerardo Perez, Galt P Barber, Anna Benet-Pages, Jonathan Casper, Hiram Clawson, Mark Diekhans, Clay Fischer, Jairo Navarro Gonzalez, Angie S Hinrichs, Christopher M Lee, Luis R Nassar, Brian J Raney, Matthew L Speir, Marijke J van Baren, Charles J Vaske, David Haussler, W James Kent, Maximilian Haeussler

**Affiliations:** Genomics Institute, University of California Santa Cruz, Santa Cruz, CA 95064, USA; Genomics Institute, University of California Santa Cruz, Santa Cruz, CA 95064, USA; Institute of Neurogenomics, Helmholtz Zentrum Munchen GmbH - German Research Center for Environmental Health, 85764 Neuherberg, Germany; Medical Genetics Center[Medizinisch Genetisches Zentrum), Munich 80335, Germany; Genomics Institute, University of California Santa Cruz, Santa Cruz, CA 95064, USA; Genomics Institute, University of California Santa Cruz, Santa Cruz, CA 95064, USA; Genomics Institute, University of California Santa Cruz, Santa Cruz, CA 95064, USA; Genomics Institute, University of California Santa Cruz, Santa Cruz, CA 95064, USA; Genomics Institute, University of California Santa Cruz, Santa Cruz, CA 95064, USA; Genomics Institute, University of California Santa Cruz, Santa Cruz, CA 95064, USA; Genomics Institute, University of California Santa Cruz, Santa Cruz, CA 95064, USA; Genomics Institute, University of California Santa Cruz, Santa Cruz, CA 95064, USA; Genomics Institute, University of California Santa Cruz, Santa Cruz, CA 95064, USA; Genomics Institute, University of California Santa Cruz, Santa Cruz, CA 95064, USA; Genomics Institute, University of California Santa Cruz, Santa Cruz, CA 95064, USA; Genomics Institute, University of California Santa Cruz, Santa Cruz, CA 95064, USA; Genomics Institute, University of California Santa Cruz, Santa Cruz, CA 95064, USA; Genomics Institute, University of California Santa Cruz, Santa Cruz, CA 95064, USA; Genomics Institute, University of California Santa Cruz, Santa Cruz, CA 95064, USA

## Abstract

The UCSC Genome Browser (https://genome.ucsc.edu) is a widely utilized web-based tool for visualization and analysis of genomic data, encompassing over 4000 assemblies from diverse organisms. Since its release in 2001, it has become an essential resource for genomics and bioinformatics research. Annotation data available on Genome Browser includes both internally created and maintained tracks as well as custom tracks and track hubs provided by the research community. This last year's updates include over 25 new annotation tracks such as the gnomAD 4.1 track on the human GRCh38/hg38 assembly, the addition of three new public hubs, and significant expansions to the Genome Archive[GenArk) system for interacting with the enormous variety of assemblies. We have also made improvements to our interface, including updates to the browser graphic page, such as a new popup dialog feature that now displays item details without requiring navigation away from the main Genome Browser page. GenePred tracks have been upgraded with right-click options for zooming and precise navigation, along with enhanced mouseOver functions. Additional improvements include a new grouping feature for track hubs and hub description info links. A new tutorial focusing on Clinical Genetics has also been added to the UCSC Genome Browser.

## Graphical Abstract

**Graphical Abstract:**
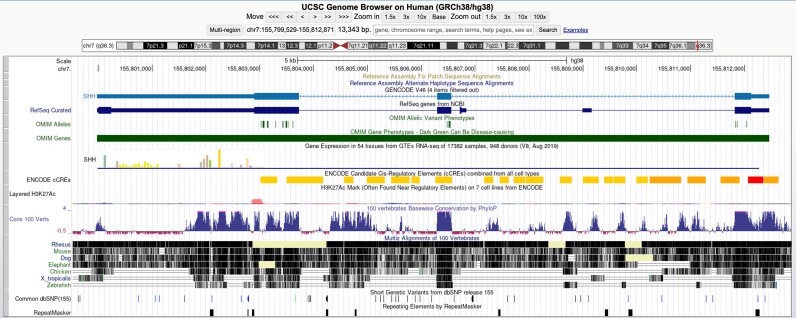

## Introduction

The University of California Santa Cruz[UCSC) Genome Browser is an interactive web tool that allows for the visualization, retrieval, and analysis of genomic data from a wide range of organisms. Launched in 2001, the UCSC Genome Browser initially provided access to a single draft genome assembly (1). Over the years, it has significantly expanded and now hosts over 4000 assemblies, encompassing data from numerous species. Users can access specific genome assemblies through the Gateway page by entering the assembly name or the GC accession number identifier from GenBank (2). Users can also search for and request specific assemblies that may not be readily available in the existing database by using the Assembly Request page (https://genome.ucsc.edu/assemblyRequest.html).

The UCSC Genome Browser's most frequently used tool provides a visual display of datasets that have been aligned to an assembly sequence; displayed datasets are commonly referred to as tracks. The most popular assemblies among our users, human GRCh38/hg38 and GRCh37/hg19, feature over 37 000 data tracks. Our next most popular assemblies, for mice, provide over 9000 additional data tracks (3). Tracks are organized into several categories: Gene, Regulation, Variation, and others to provide a structured way to navigate the vast amount of data available. To further aid users in navigating this extensive data collection, the UCSC Genome Browser offers a Track Search feature. This feature allows users to input search terms that query track descriptions, group classifications, and track names within a selected assembly and receive back a list of relevant datasets. Users can also upload their data to the UCSC Genome Browser through custom tracks or track hubs to visualize those data alongside the natively available tracks. Beyond visualization, the UCSC Genome Browser also offers both web-based and command-line tools with over hundreds of utilities that can assist users with data analysis.

The UCSC Genome Browser is accessible via the primary UCSC site (https://genome.ucsc.edu) and can also be accessed through our European (https://genome-euro.ucsc.edu) and Asian (https://genome-asia.ucsc.edu) mirror sites. These mirror sites ensure reliable and fast access to the UCSC Genome Browser resources worldwide, catering to an international user base; serving over 7000 unique users daily, with an estimated annual user base of 1.4 million. For users whose requirements are incompatible with accessing a web server hosted externally, such as when working with protected or embargoed data, we offer alternative solutions. The Genome Browser in the Cloud (GBiC) installation script enables the setup of a full mirror of the UCSC Genome Browser on the user's own server or cloud infrastructure. Additionally, the Genome Browser in a Box (GBiB) provides a virtual machine version of the UCSC Genome Browser, designed to run locally on a laptop or desktop computer. Both options ensure that protected data remain securely within the user's environment without being transmitted to UCSC.

The UCSC Genome Browser is continually updated to incorporate new features and data. Software updates are released on a tri-weekly basis, accompanied by announcements detailing new features, track releases, and other updates. This regular update schedule ensures that the software remains at the forefront of genomic research tools, continually offering the latest data and functionality to its users.

### New and updated annotations

Over the past year, the UCSC Genome Browser has undergone significant updates and expansions. Notably, more than 25 tracks have been added or updated across various assemblies. This includes both the addition of new clinical tracks, which provide vital information relevant to medical and translational research, as well as updates to existing gene tracks to ensure they reflect the latest genomic annotations and discoveries. We have also introduced three new public hubs.

### New clinical tracks

This year, ten new clinical tracks were incorporated into our human assemblies. Among these, the gnomAD 4.1 track stands out; it presents variants from 807 162 individuals, including 730 947 exomes and 76 215 genomes (4). Additionally, we have introduced new prediction score tracks, including AbSplice, BayesDel, and Illumina SpliceAI. AbSplice is a method that predicts aberrant splicing across human tissues. Its track displays precomputed AbSplice scores for all possible single-nucleotide variants genome-wide (5). BayesDel provides a deleteriousness meta-score for coding and non-coding variants, single nucleotide variants and small insertions/deletions (6). SpliceAI is an open-source deep learning splicing prediction algorithm that can predict splicing alterations caused by DNA variations (7).

Another significant addition to our human assemblies is the DECIPHER Dosage Sensitivity track. This track utilizes an ensemble machine learning model to predict dosage sensitivity probabilities (pHaplo & pTriplo) for all autosomal genes. It has identified 2 987 haploinsufficient and 1 559 triplosensitive genes, including 648 genes uniquely identified as triplosensitive (8).

### Gene set updates

Six gene tracks have been added and updated in both human and mouse assemblies, including the GENCODE KnownGene tracks, now updated to version 46 for human (V46) and version VM35 for mouse (9). The GENCODE ‘KnownGene’ v45lift37 gene track has also been integrated for the hg19 assembly, along with the GENCODE/UCSC Genes Archive superTrack, which contains the 2013 UCSC Genes track for reproducibility purposes. The GENCODE KnownGene tracks serve as the default gene tracks for human and mouse assemblies, with each gene in these tracks is associated with metadata and corresponding records at other resources.

A RefSeq Historical track has been added for the hg38 assembly, enabling the search of previous RefSeq transcript versions, including NM_ accessions and HGVS searches (10,11). We have also included gene tracks that undergo annual automatic updates, such as the HGMD and MANE tracks for human assemblies. The HGMD track displays transcripts with clinical variants from the Human Gene Mutation Database (HGMD) (12). The Matched Annotation from NCBI and EMBL-EBI (MANE) track showcases high-confidence transcripts that are identically annotated between RefSeq (NCBI) and Ensembl/GENCODE (led by EMBL-EBI) (13).

**Figure 1.**
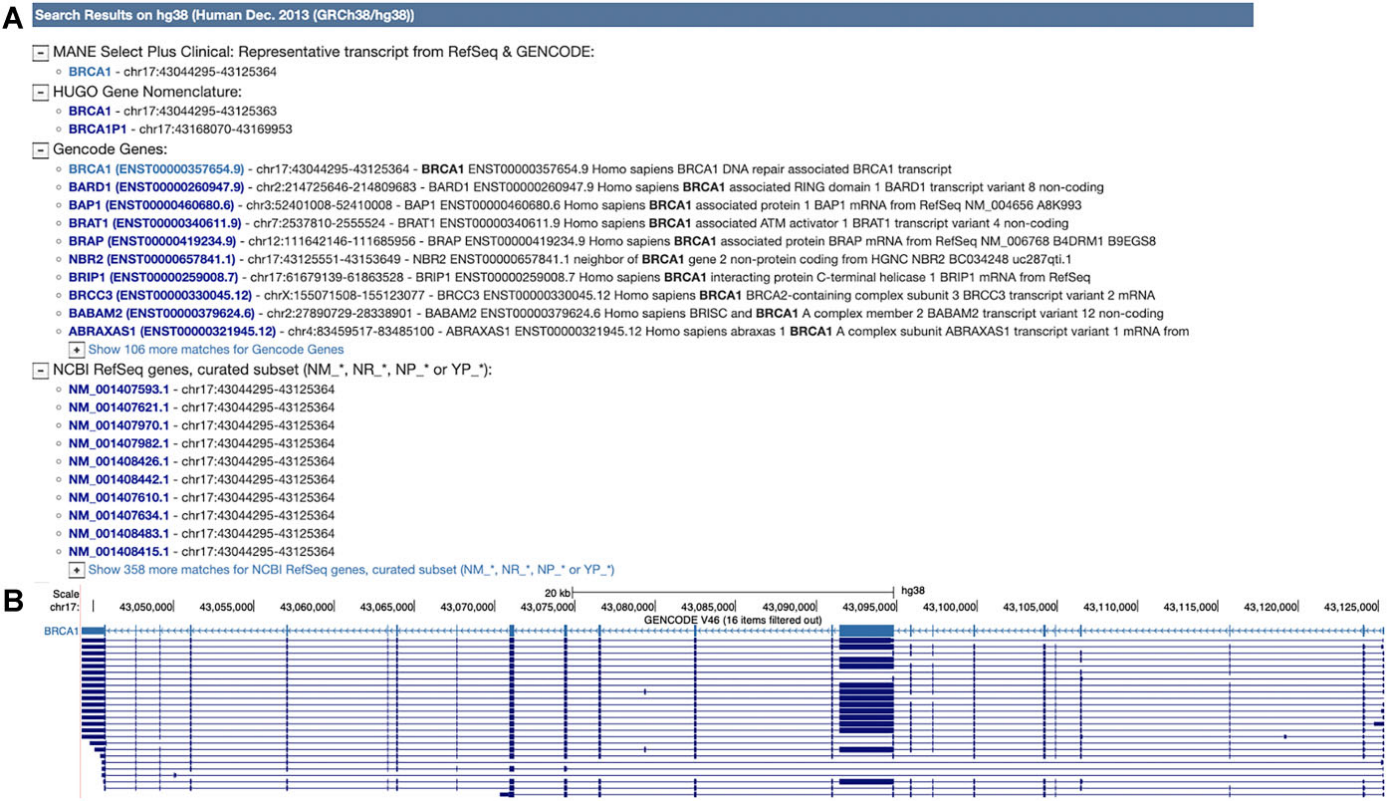
(**A**) MANE transcripts displayed as the top result. (**B**) MANE transcript color for the tumor suppressor ‘BRCA1’ gene in the Genome Browser display.

In addition, the search results have been enhanced to prioritize MANE transcripts, which are now displayed as the top result (Figure 1A). The color of the MANE transcripts has also been updated to distinguish them from other transcripts (Figure 1B).

### Other new tracks

In addition, we have added and updated 10 new tracks to our vertebrate assemblies. Among these, the CRISPR Targets track for the Telomere-to-Telomere assembly (hs1 human) identifies DNA sequences that can be targeted by CRISPR RNA guides using the Cas9 enzyme from *S. pyogenes* (PAM: NGG) (14). The VISTA Enhancers track has been incorporated into both human and mouse assemblies, displaying potential enhancers whose activity has been experimentally validated in transgenic mice (15).

We have also added the EVA SNP release 6 for 37 assemblies, which includes mappings of single nucleotide variants and small insertions and deletions (indels) (16). Furthermore, the Variants of Concern track has been updated to include the latest WHO-designated variants of concern (VOC), highlighting amino acid and nucleotide mutations in SARS-CoV-2 variants as defined in December 2021 (17).

### New hubs

We accept submissions of datasets or assemblies to be featured as ‘Public hubs’. These track hubs are announced upon their addition to the public hubs page. This year, we have introduced three new public hubs.

The first new public hub is the ImpactHub, which utilizes the Impact machine learning model to provide predictions across 707 pairs of transcription factors (TFs) and cell types for regulatory element activity (18). The second is the Predominant PAS hub, which displays the locations of predominant polyadenylation sites and predominant polyadenylation hexamers for almost 16 000 protein-coding genes (19). The third hub is an assembly hub featuring the Sunflower Sea Star (Pycnopodia helianthoides) (20). These hubs are maintained by their respective authors.

### Genome Archive (GenArk)

We continue to expand our GenArk hub library, adding over 1000 new GenArk assemblies, each equipped with Genome Browser annotations and BLAT support. This expansion includes the addition of Telomere-to-Telomere (T2T) primate and mouse assemblies to GenArk. Furthermore, we have integrated IGV outlinks from GenArk index pages to enhance usability and data access.

Recently, we published a new paper titled ‘GenArk: Towards a Million UCSC Genome Browsers,’ which details our Genome Archive (GenArk) system and its ongoing development (21).

### New genome browser software

We have updated our software, particularly enhancing the visualization of our pages. The browser graphic page has undergone several modifications aimed at improving the user experience.

### Browser graphic page

**Figure 2.**
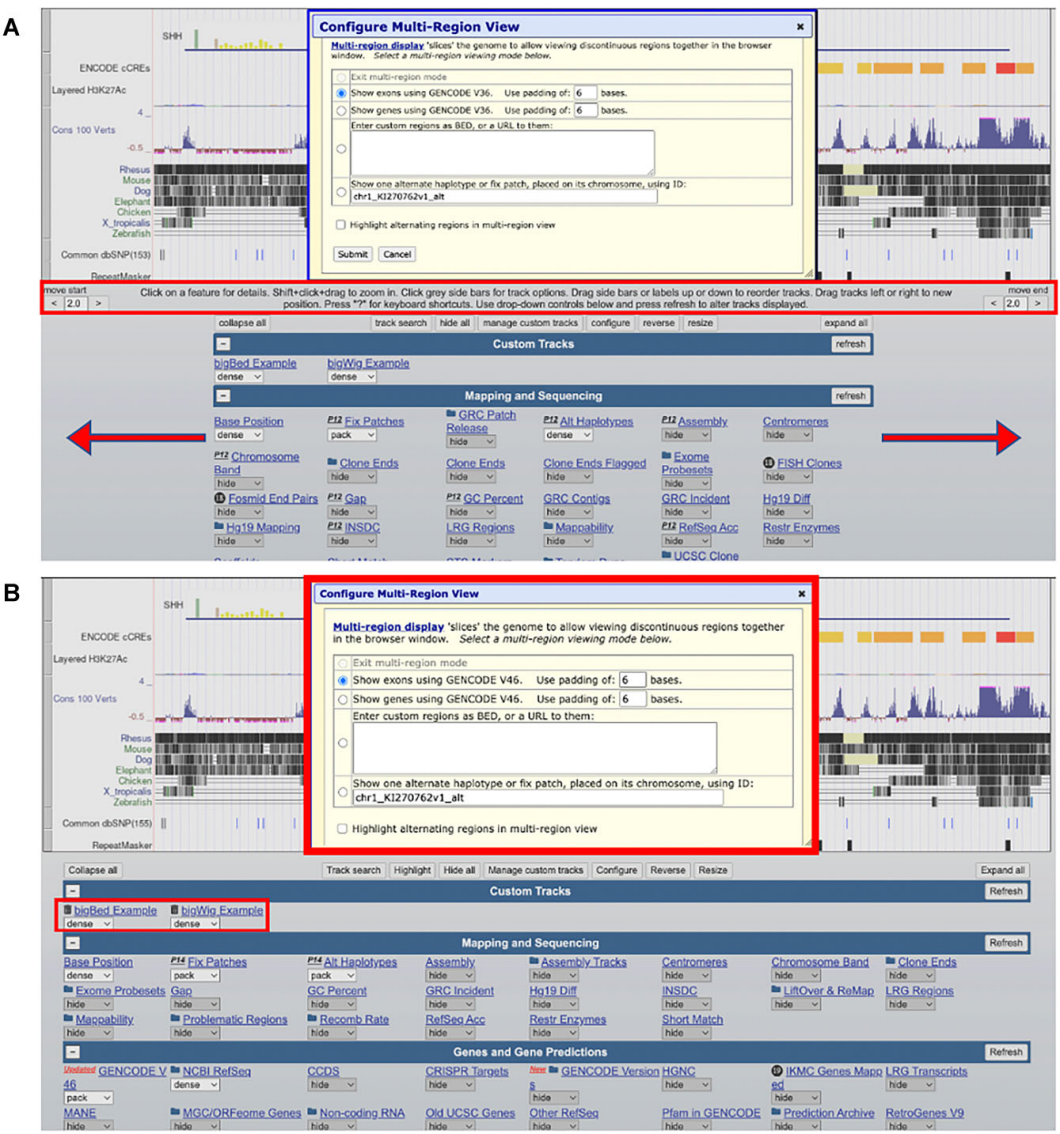
(**A**) previous browser graphic page. (**B**) current browser graphic page displays trash icons next to custom tracks, more track columns on wider screens, increased font sizes for dialog boxes, and a reduction in text on the page.

The browser graphic page displays data annotations, known as tracks, for a reference genome. Users can zoom, drag, and configure these display annotations. The browser graphic page has undergone several updates, the addition of trash icons next to custom tracks for quick removal, enhanced the display for more track columns on wider screens, increased font sizes for dialog boxes, and reduction in text amount on the page (Figure 2).

**Figure 3.**
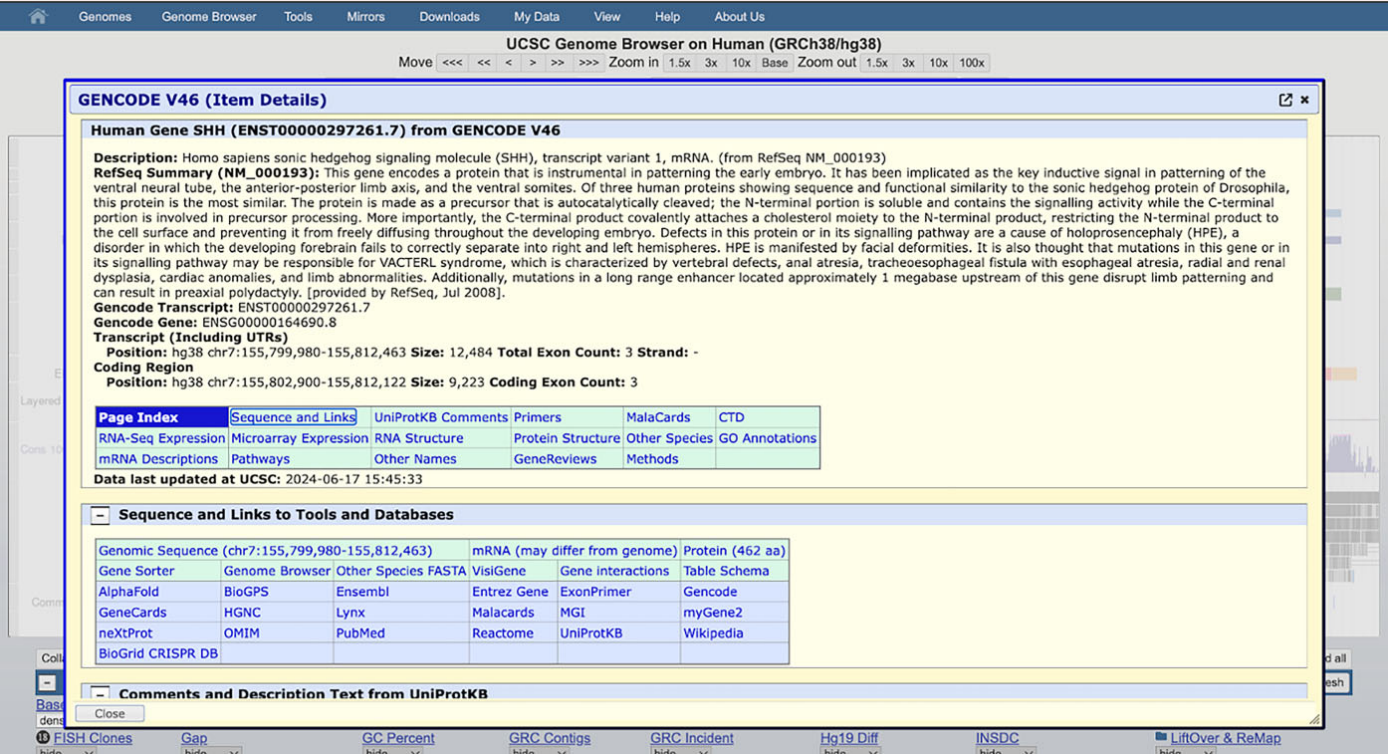
The new popup dialog box that shows item details for the hg38 GENCODE ‘KnownGene’ track.

We have introduced the new Item Details feature which simplifies the user experience by displaying track item details in a pop-up dialogue box. This feature allows the information to be viewed without the need to navigate away from the current page (Figure 3).

**Figure 4.**
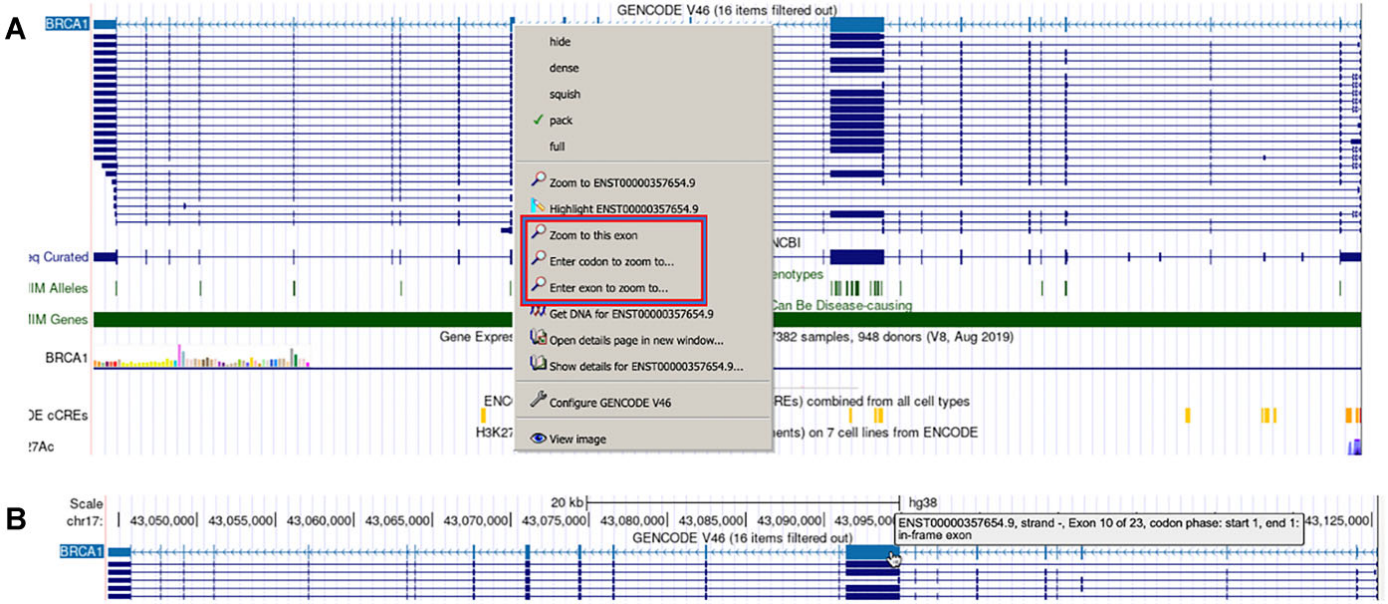
(**A**) Right-click options for genePred tracks. (**B**) Exon mouse over display for genePred tracks.

The genePred tracks, such as the GENCODE and NCBI RefSeq tracks, have undergone several updates. By right-clicking on a genePred track, users now have options for zooming, entering an exon position, or entering a codon for quicker navigation within the browser graphic (Figure 4A). The mouseOver function in genePred tracks has also been enhanced to display the phase of the first and last codon. Additionally, it now indicates whether exons are in-frame or out-of-frame (Figure 4B).

**Figure 5.**
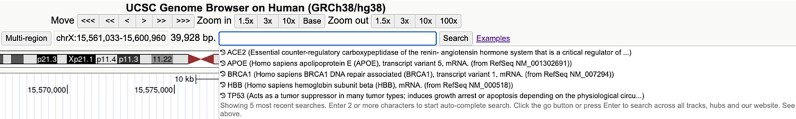
Search box shows the five most recent search terms.

The search box on the browser graphic display allows users to enter position queries or find terms that match track data, track descriptions, help documents, and public hub track descriptions. We have updated the search box which now shows the five most recent search terms. Any genes searched within the browser's graphical display or terms selected from the search results page will be displayed beneath the search box (Figure 5).

### New hub features

**Figure 6.**
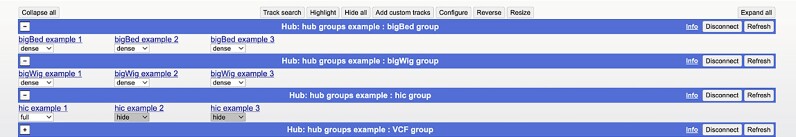
Grouping of track hubs on the browser graphic page.

We have introduced a new grouping feature for track hubs, which allows the structured organization of tracks into distinct groups (Figure 6). This can reduce the need for multiple hubs, each requiring separate files, by consolidating tracks into grouped hubs, where each group is managed within a single hub. This feature can be applied to a UCSC genome, a GenArk assembly, or an assembly hub. These track hub groups are kept separate from other track hubs and the native UCSC Genome Browser track groups, allowing for greater organizational flexibility. For instance, you can add a ‘genes’ group without causing conflicts or confusion.

**Figure 7.**
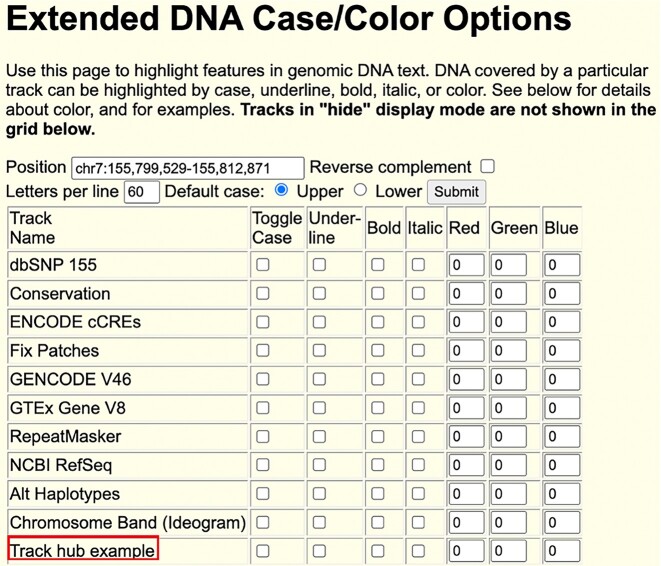
Extended DNA case/color options for a track hub.

Additionally, we have added an info link to the track hub's blue bar name, directing users to the hub description page for detailed information about the track hub. We have also implemented the highlighting of genomic DNA covered by track hubs using the Extended DNA Case/Color Options found under View → DNA Sequence (Figure 7). Highlighting options include case changes, underlining, bold, italics or color.

### New and updated tools

We offer a REST API that enables querying of both annotation and sequence data from any UCSC genome assembly or hub (22). This year, we have expanded API support to include bigChain, bigMaf and bigDbSnp track types. Additionally, we introduced a new API function, revComp, which retrieves the reverse complement of a given sequence.

### Tutorial

We previously offered an interactive introduction tutorial for new users and have now added a new tutorial specifically focused on Clinical Genetics in the UCSC Genome Browser. This clinical tutorial guides users on searching for variants and related queries using HGVS terms, genome coordinates, gene symbols, and specific annotation IDs like NM identifiers and rsIDs. It also explains how to find recommended track sets that help configure displays with relevant annotations for variant interpretation. It includes examples of selecting tracks such as the Clinical SNVs and Clinical CNVs track sets. The tutorial highlights other features that may assist in variant interpretation.

### Email support

We continue to offer email support through both public and confidential mailing lists, where UCSC Genome Browser staff address questions regarding tools or data. More details about our mailing lists can be found at https://genome.ucsc.edu/contacts.html, including a link to the public list, which archives previously answered questions.

### Future plans

A major goal is to implement user accounts with 10 GB of dedicated storage for uploading annotation files and attaching them as track hubs. As storage costs decrease, we anticipate that future upgrades to our storage array will provide significantly larger capacity for the same cost as our current system. Additionally, we aim to offer a hub maker interface tool for files uploaded into a user's storage space, facilitating the creation of basic hubs. We continue to make progress on the Liftover on the Fly feature, which enables the automatic lifting of annotations to unannotated genomes without the need for manual track lifting. Furthermore, we are developing more beginner-friendly tutorials, which will include guides on the Gateway page, UCSC Genome Browser graphic display, and the Table Browser.

## Data Availability

All users can use the UCSC Genome Browser (https://genome.ucsc.edu/) freely but with exceptions to the source code for the Blat utility, liftOver utility and other utilities which are free for non-profit academic research and for personal use. A license is required for commercial use of these utilities or the source code.

## Notes

Present address: Gerardo Perez, Genomics Institute, University of California Santa Cruz, 1156 High Street, Santa Cruz, CA 95064, USA.
